# Comparative Proteomics of Plasma and Flagellar Membranes in *Chlamydomonas reinhardtii*

**DOI:** 10.3390/ijms27146141

**Published:** 2026-07-09

**Authors:** Yiwen Lin, Sheng Yao, Huan Long, Kaiyao Huang

**Affiliations:** 1 Key Laboratory of Algal Biology, Institute of Hydrobiology, Chinese Academy of Sciences, Wuhan 430072, China; linyw@ihb.ac.cn; 2 College of Life Sciences, University of Chinese Academy of Sciences, Beijing 100039, China; 3 Key Laboratory of Pesticide & Chemical Biology of Ministry of Education, Hubei Key Laboratory of Genetic Regulation and Integrative Biology, School of Life Sciences, Central China Normal University, Wuhan 430079, China; y_sheng@mails.ccnu.edu.cn

**Keywords:** *Chlamydomonas reinhardtii*, flagellar membrane, plasma membrane, comparative proteomics, flagella

## Abstract

The flagellar (ciliary) membrane of *Chlamydomonas reinhardtii* is continuous with the plasma membrane but maintains a distinct protein composition through the ciliary gate mechanism; however, a systematic comparison of the membrane proteomes of these two compartments has not been reported. Here, we performed comparative proteomics on highly enriched plasma and flagellar membrane fractions and identified 2779 proteins, with hundreds differentially abundant. Plasma membrane-enriched proteins were mainly involved in proton transport and ATP synthesis, whereas flagellar membrane-enriched proteins were specialized for cilium movement, axoneme assembly, and intraflagellar transport. Fluorescence localization validated the proteomic data and revealed previously uncharacterized subcellular patterns. Notably, PLD1 showed dual localization at the plasma membrane and punctate cytoplasmic structures, and FAP215 (validated by subflagellar fractionation) displayed a broader flagellar signal than the central microtubule protein FAP178, consistent with its localization in the flagellar matrix or membrane. This work provides the first systematic comparison of plasma and flagellar membrane proteomes in *Chlamydomonas*, offering a valuable resource for understanding flagellar membrane specialization.

## 1. Introduction

The unicellular green alga *Chlamydomonas reinhardtii* has long served as a powerful model organism for studying the biology of cilia and flagella [1,2,3,4]. Its unique experimental advantages, including the ability to detach and isolate flagella in a single step without cell lysis, have made it an unparalleled system for dissecting the composition, assembly and function of these evolutionarily conserved organelles [5,6].

The flagellar membrane is a specialized subdomain of the plasma membrane [7,8,9]. Although it is continuous with the surrounding plasma membrane, it maintains a distinct protein [10,11,12] and lipid composition [13,14] essential for its sensory, secretion and signaling [15,16,17,18,19,20] and motile functions [21,22,23,24,25]. This compositional uniqueness is established and preserved by a “ciliary gate” located at the base of the flagellum, which functions as a diffusion barrier that restricts the free exchange of membrane proteins between the flagellar and somatic compartments [5,26,27,28]. Identifying the specific proteins that reside in the flagellar membrane versus those confined to the surrounding plasma membrane is therefore critical for understanding how this gate operates and how flagella execute their specialized functions.

Substantial progress has been made in characterizing each membrane system independently. Pazour and colleagues performed the first high-confidence proteomic analysis of *C. reinhardtii* flagella, identifying hundreds of proteins, many of which are membrane-associated components of motor and signaling pathways [12]. Subsequently, our group developed a method for isolating flagellar membrane vesicles and demonstrated that the flagellar membrane and its secreted ectosomes possess distinct protein compositions [16]. This technical advance enabled us to further compare the protein composition of the flagellar membrane with that of the flagellar vesicles [16]. In parallel, robust methods for purifying *C. reinhardtii* plasma membranes have been established, most notably using aqueous two-phase partitioning [29,30,31]. However, despite these independent advances, the plasma membrane and the flagellar membrane have never been analyzed together within a single, directly comparative proteomic framework. The current knowledge gap lies in bridging these datasets—generated using different strains, conditions and analytical pipelines—to quantitatively assess the genuine differences in membrane protein composition.

In this study, we addressed this gap by establishing a comprehensive comparative proteomics pipeline. Building on our previous experience in flagellar membrane isolation [16], we systematically established optimized protocols for the isolation of highly enriched plasma membranes and flagellar membranes from *C. reinhardtii* as a prerequisite for the comparative proteomic analysis. Using these fractions, we performed the first direct comparative proteomics to identify proteins specifically enriched in each compartment, and supported our quantitative findings through independent fluorescence localization. By providing the first direct comparison of these two continuous yet functionally distinct membrane systems, this work defines the molecular identity of the flagellar membrane proteome in relation to the plasma membrane and establishes a foundational resource for future studies on ciliary membrane specialization, and the mechanisms of ciliary protein sorting.

## 2. Results

### 2.1. Isolation and Purity Validation of Plasma Membrane

**Figure 1:**
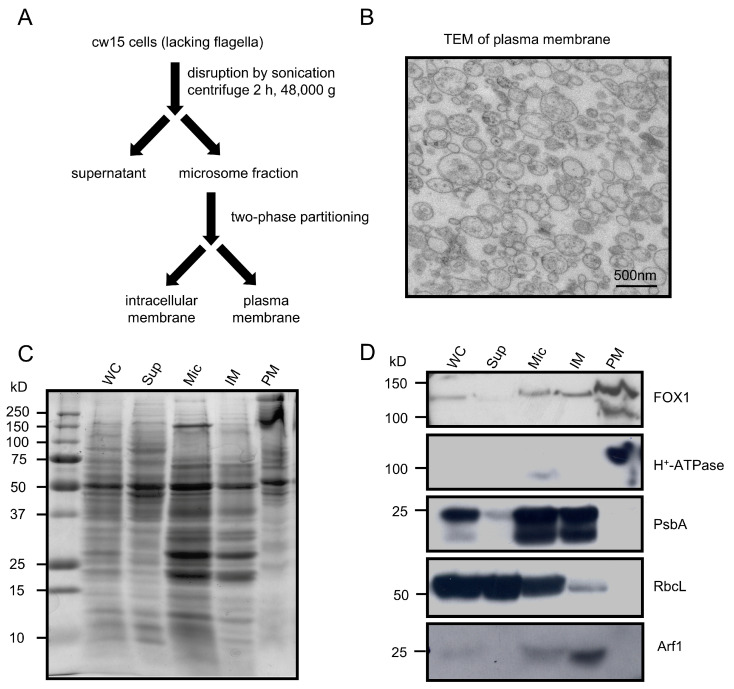
Isolation and purity validation of plasma membrane. (**A**) Schematic workflow of plasma membrane (PM) isolation. Cell wall-deficient *cw15* cells were disrupted by sonication, followed by differential centrifugation to obtain a microsome pellet. The microsomes were then subjected to aqueous two-phase partitioning (6.4% dextran T500/6.4% PEG 4000). The upper PEG-rich phase, containing PM vesicles, was collected and pelleted by ultracentrifugation. (**B**) Transmission electron micrograph (TEM) of negatively stained PM vesicles (scale bar = 500 nm). (**C**) Coomassie Blue-stained SDS-PAGE (10 µg protein/lane) of fractions collected during isolation. The distinct banding pattern of the PM fraction indicates effective removal of non-PM proteins. (**D**) Western blot of the same fractions. PM markers FOX1 (~120 kDa) and H^+^-ATPase (~109 kDa) were enriched in PM; chloroplast markers PsbA (24 kDa & 10 kDa) and RbcL (~52 kDa) and Golgi marker Arf1 (~21 kDa) were undetectable, confirming highly enriched target markers. (**C**,**D**) Fraction abbreviations: WC, whole cells; Sup, supernatant; Mic, microsome; IM, intracellular membranes; PM, plasma membrane.

To obtain highly enriched plasma membrane (PM) fractions for comparative proteomics, we applied an aqueous two-phase partitioning method (Figure 1A). The cell-wall-deficient *cw15* strain [32] was used to facilitate efficient cell lysis and recovery of intact membrane vesicles.

Transmission electron microscopy of negatively stained PM vesicles revealed a homogeneous population of round to oval vesicles with clean boundaries and diameters mainly ranging from 100 to 500 nm, indicating the integrity and high enrichment of the preparation (Figure 1B). Polypeptide profiles of the fractions collected throughout the isolation were visualized by Coomassie Brilliant Blue staining (Figure 1C). Whole cell lysate displayed a complex banding pattern across the entire molecular weight range. In striking contrast, the final PM fraction presented a markedly different protein profile. Notably, the PM lane showed very few bands in the 15–25 kDa region, where the microsome and intracellular membrane fractions displayed abundant low-molecular-weight proteins. This significant difference indicates that the two-phase partitioning substantially removed a large portion of low-molecular-weight non-PM proteins, yielding a highly enriched PM preparation with a distinct polypeptide composition.

To assess cross-contamination from other organelles, we probed the same fractions with compartment-specific antibodies (Figure 1D). As expected, the PM markers FOX1 [29] and H^+^-ATPase [12,30] were strongly enriched in the final PM fraction. In contrast, the chloroplast markers PsbA (D1 protein of photosystem II) and RbcL (Rubisco large subunit) [33] were undetectable in the PM fraction. Similarly, the Golgi/endosomal marker Arf1 [34] was absent from the purified PM. These results indicate that the isolated PM fraction is highly enriched and devoid of detectable contamination by chloroplast, thylakoid, and endomembrane systems.

### 2.2. Isolation and Purity Validation of Flagellar Membrane

**Figure 2:**
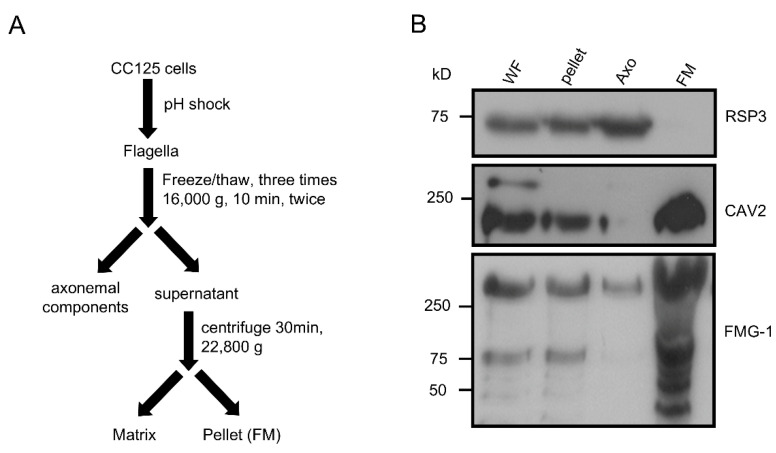
Isolation and purity validation of flagellar membrane. (**A**) Schematic workflow of flagellar membrane (FM) isolation. Flagella were detached from CC125 cells by pH shock, purified through a sucrose cushion, and collected by centrifugation. Purified flagella were subjected to three freeze–thaw cycles, followed by low-speed centrifugation to remove axonemes, and high-speed centrifugation to pellet the FM fraction. (**B**) Western blot of fractions (whole flagella, pellet, axoneme, FM) using compartment-specific markers. Axonemal marker RSP3 (~70 kDa) was detected only in the axoneme fraction; flagellar membrane markers CAV2 (~220 kDa) and FMG-1 (a high-molecular-weight smear at ~350 kDa, typically accompanied by lower molecular weight bands likely representing proteolytic breakdown products) were enriched in FM. WF, whole flagella; Axo, axoneme; FM, flagellar membrane.

Since the cell wall-deficient *cw15* strain [32] is highly susceptible to mechanical lysis, it facilitates gentle lysis and intact PM vesicle recovery; by contrast, the wild-type CC-125 has an intact wall and normal flagella [35], and is commonly used for isolating flagella. Flagellar membranes (FM) were isolated from wild-type *C. reinhardtii* (CC-125) using a pH-shock deflagellation method [36] followed by sucrose cushion purification and liquid nitrogen freeze–thaw lysis (Figure 2A). Briefly, detached flagella were purified through a sucrose cushion, then subjected to three cycles of liquid nitrogen freeze–thaw to disrupt the flagellar membrane. After removal of axonemal components by low-speed centrifugation (16,000× *g*, twice), the flagellar membrane was pelleted by high-speed centrifugation (22,800× *g*).

To assess the enrichment of the flagellar membrane preparation, we examined whole flagella, the pellet after freeze–thaw lysis (axoneme-enriched), and the final flagellar membrane fraction with compartment-specific antibodies (Figure 2B). The axonemal marker RSP3 (radial spoke protein 3) [37] was abundant in whole flagella and strongly enriched in the axoneme pellet, but was undetectable in the flagellar membrane fraction, confirming efficient removal of axonemal components. Conversely, the flagellar membrane markers CAV2 (voltage-dependent Ca^2+^ channel) [38] and FMG-1 [39,40] were present in whole flagella and strongly enriched in the flagellar membrane fraction, with only trace signals remaining in the axoneme pellet. These results indicate that the isolated flagellar membrane fraction is highly enriched for flagellar membrane proteins and devoid of detectable axonemal contaminants, making it suitable for subsequent comparative proteomic analysis.

### 2.3. Comparative Characterization of PM and FM Fractions

**Figure 3:**
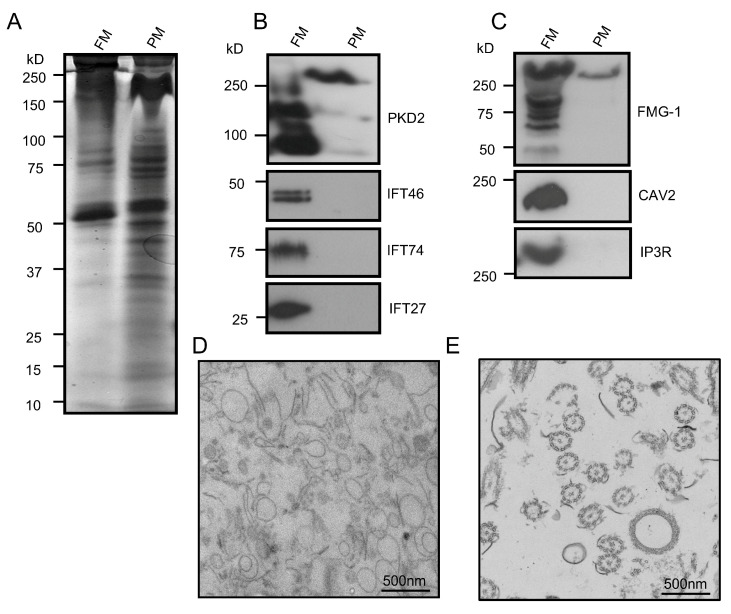
Biochemical and ultrastructural characterization of plasma membrane and flagellar membrane. (**A**) Coomassie Blue-stained SDS-PAGE (10 µg/lane) of plasma membrane (PM) and flagellar membrane (FM). Distinct banding patterns indicate different protein compositions. (**B**,**C**) Western blot of PM and FM using compartment-specific antibodies. (**B**) Flagellar markers PKD2 (120 and 90 kDa), IFT46, IFT74 and IFT27 were detected only in FM. (**C**) FM markers FMG-1 (~350 kDa smear) and CAV2 (~220 kDa) were enriched in FM; IP3R (~300 kDa) was also enriched in FM. (**D**) TEM of negatively stained PM vesicles (scale bar = 500 nm). Uniform vesicles with clean boundaries. (**E**) TEM of isolated axonemes after membrane removal, showing “9 + 2” microtubule arrangement (scale bar = 500 nm). These data indicate the high enrichment and integrity of both membrane fractions.

To directly compare the protein composition and enrichment of the two membrane fractions, we performed parallel SDS-PAGE, Western blotting, and transmission electron microscopy on the final PM and FM preparations (Figure 3).

The total protein profiles of PM and FM were visualized by Coomassie Brilliant Blue staining (Figure 3A). The two lanes displayed markedly distinct banding patterns, indicating that the two membrane fractions differ substantially in their protein composition. Notably, the PM lane exhibited a prominent cluster of bands between 75 and 250 kDa (corresponding to PM markers such as FOX1 and H^+^-ATPase), whereas the FM lane lacked these bands but showed a characteristic high-molecular-weight smear in the ~350 kDa region, which corresponds to the heavily glycosylated flagellar membrane protein FMG-1 as confirmed by Western blot (Figure 3C). Conversely, the FM lane contained very few bands below 50 kDa, in contrast to the PM lane which had more low-molecular-weight bands (Figure 3A).

To quantitatively assess the enrichment of specific markers and exclude cross-contamination, we probed the PM and FM fractions with a panel of compartment-specific antibodies (Figure 3B,C). As expected, the flagellar membrane marker PKD2 [39], detected mainly as bands of 120 and 90 kDa, was present in the flagellar membrane fraction. The higher molecular mass band detected in the PM fraction likely represents the full-length 210 kDa precursor of PKD2, which is known to localize to the cell body (including the ER) and undergoes proteolytic processing to generate the active 120 kDa and 90 kDa fragments that are selectively enriched in flagella [39,41,42]. The flagellar proteins IFT46, IFT74 and IFT27 were also detected exclusively in this fraction [12]. The flagellar membrane markers FMG-1 [39,40], CAV2 [38] and IP3R [16] were specifically enriched in the flagellar membrane fraction [12,30]. These results confirm the substantial enrichment of the flagellar membrane markers in the FM fraction, consistent with the purity validation shown for the PM fraction (Figure 1D) and the FM fraction (Figure 2B). Together, these data support that both membrane fractions are substantially enriched for their respective markers.

Transmission electron microscopy of the isolated PM revealed uniform, round to oval vesicles with clean boundaries (Figure 3D), while the FM-depleted axonemes obtained after freeze–thaw and centrifugation displayed typical “9 + 2” microtubule arrays (Figure 3E), confirming the integrity of the axoneme after membrane removal. Together, the biochemical and morphological data indicate the substantial enrichment and distinct protein composition of the PM and FM fractions, providing a solid foundation for comparative proteomics.

### 2.4. Comparative Proteomic Analysis of PM and FM

To comprehensively characterize the protein composition of the two membrane fractions, we extracted protein components from the plasma membrane and flagellar membrane and performed labeled quantitative proteomic identification. The MS/MS spectrum database search analysis identified a total of 2779 proteins, of which 2287 were quantifiable (Table 1 and Table S1). This high-coverage dataset provides a robust basis for comparative analysis.

**Figure 4:**
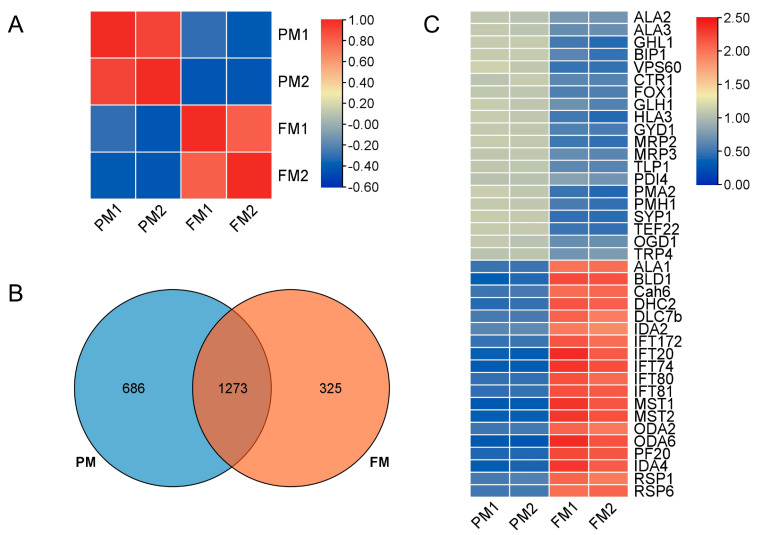
Comparative proteomic analysis of plasma membrane (PM) and flagellar membrane (FM). (**A**) Pearson correlation heatmap of all samples (PM-1, PM-2, FM-1, FM-2). The color scale indicates correlation coefficients (red, r = 1; white, r = 0; blue, r = −1). (**B**) Venn diagram showing the distribution of all identified quantifiable proteins between PM and FM fractions. An intensity threshold (original intensity > 5) was used as a criterion to determine whether a protein was reliably detected in a given fraction. Three proteins with intensities below the threshold in both fractions were not assigned to either category. (**C**) Heatmap of 40 representative membrane-associated marker proteins, comprising 20 known PM markers and 20 known FM markers. Red: higher abundance; blue: lower abundance. PM and FM replicates clearly separate, confirming the distinct protein compositions of the two membrane fractions.

To evaluate the overall similarity and reproducibility among samples, we performed Pearson correlation analysis on the four biological replicates (Figure 4A). The correlation coefficient between the two PM replicates (PM-1 vs. PM-2) was 0.98, and that between the two FM replicates (FM-1 vs. FM-2) was 0.97, indicating excellent technical and biological reproducibility. In contrast, cross-comparisons between PM and FM samples showed negative correlations (ranging from −0.87 to −0.82), confirming that the two membrane fractions possess distinct proteomic profiles.

Since a simple presence/absence Venn diagram can be misleading due to the inclusion of low-abundance, low-confidence protein identifications, we applied an abundance-based filter (original intensity > 5) to focus on high-confidence proteins. After filtering, the Venn diagram (Figure 4B) revealed 686 proteins uniquely detected in PM, 325 proteins uniquely detected in FM, and 1273 proteins shared between the two fractions. These numbers indicate that the two membrane-enriched preparations have substantially different protein compositions when only high-abundance proteins are considered.

To further validate the qualitative separation, we examined the expression patterns of 40 well-characterized membrane-associated marker proteins (Figure 4C). This set included 20 known PM markers (e.g., FOX1, H^+^-ATPase) and 20 known FM markers (e.g., IFT20, IFT74, dynein chains). The heatmap shows that the two PM replicates cluster tightly together and are clearly separated from the two FM replicates, which also form a distinct cluster. The PM markers are strongly enriched in the PM fraction, whereas the FM markers are strongly enriched in the FM fraction. This pattern is fully consistent with the Pearson correlation results (Figure 4A) and with the independent Western blot validation (Figure 1D, Figure 2B and Figure 3C).

Collectively, the Pearson correlation, abundance-filtered Venn diagram, and marker-protein heatmap provide multiple lines of evidence that the PM- and FM-enriched fractions are highly reproducible and compositionally distinct, forming a solid basis for subsequent functional enrichment and network analyses.

To explore the functional specialization of each membrane system, we performed GO and KEGG pathway analyses on three groups defined solely by fold change (without relying on *p*-values): PM-enriched proteins (PM/FM ≥ 2, i.e., at least 2-fold higher in PM, Figure S1A), FM-enriched proteins (PM/FM ≤ 0.5, i.e., at least 2-fold higher in FM, Figure S1B), and non-differential proteins (0.5 < PM/FM < 2, Figure S1C).

PM-enriched proteins (Figure S1A) were significantly enriched in processes related to translation, cytoplasmic translation, and ribosomal structure. KEGG pathways included ribosome and oxidative phosphorylation, consistent with the PM’s role in protein synthesis and energy metabolism. Proton transport and ATP synthesis-related terms were also observed, reflecting PM function in ion homeostasis.

In contrast, FM-enriched proteins (Figure S1B) were strongly associated with ciliary/flagellar functions, including cilium movement, axoneme assembly, and dynein arm assembly. Enriched molecular functions included dynein chain binding and GTP binding. Cellular component terms included motile cilium, axoneme, and intraflagellar transport (IFT) particles. KEGG pathways such as phagocytosis and motor proteins were also identified. These results are consistent with the classical flagellar proteome reported by Pazour et al. [12].

Proteins shared between PM and FM (Figure S1C) showed enrichment in terms related to protein folding (e.g., unfolded protein binding, chaperone), retrograde vesicle-mediated transport, and ubiquitin-dependent proteolysis. KEGG pathways included proteasome and photosynthesis (the latter likely reflecting minor thylakoid carryover). These proteins represent core cellular housekeeping functions similarly abundant in both fractions.

### 2.5. Fluorescence Microscopy Validation Confirms the Differential Localization of Selected Membrane Proteins

**Figure 5:**
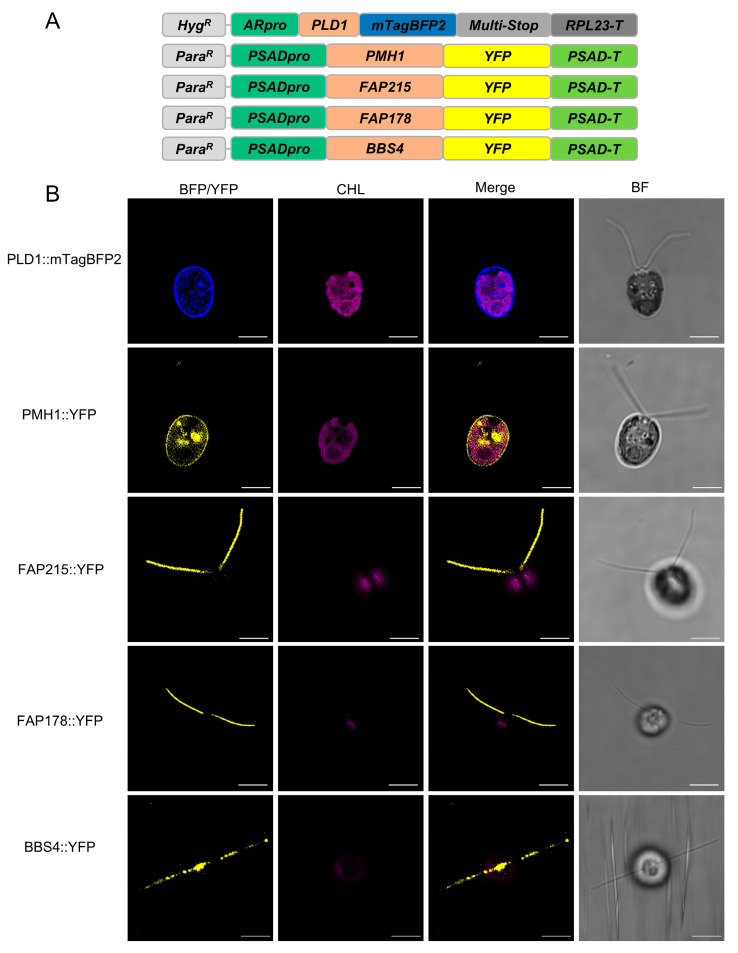
Subcellular localization validation of representative candidate proteins. (**A**) Schematic representation of expression constructs for PLD1::mTagBFP2, PMH1::YFP, FAP215::YFP, FAP178::YFP and BBS4::YFP. All constructs were introduced into *C. reinhardtii* by electroporation. (**B**) Fluorescence images of live cells expressing the indicated fusion proteins. For each construct, images show the fluorescent signal (BFP or YFP), chlorophyll autofluorescence (CHL), merge, and bright field (BF). Scale bar = 5 µm.

To independently verify the proteomic data and determine the subcellular distribution of several differentially enriched proteins, selected five candidate proteins based on their PM/FM abundance ratios and generated fluorescent fusion constructs for these representative candidates: PLD1 (putative mitochondrial cardiolipin hydrolase), PMH1 (P-type ATPase/ACA3) [43], FAP215 (novel flagellum-enriched protein), FAP178 (central microtubule protein) [44], and BBS4 (known flagellar protein) [28] (Figure 5A). Each construct was stably transformed into *C. reinhardtii* via electroporation. Expression of the fusion proteins was driven by either the *HSP70A/RBCS2* fusion promoter or *PSAD* promoter, as indicated (Figure 5A). Transformants were selected using hygromycin or paromomycin resistance markers. Live-cell imaging of the fluorescent fusion proteins was performed (Figure 5B). The temperature-sensitive *fla10* mutant [45] (CC-1919; *fla10-1* allele, encoding a subunit of the anterograde IFT motor kinesin-2, which inactivates at 32 °C) was used for subsequent functional exploration (see Discussion).

Images are representative of at least two independent transformants. Primers and construct details are provided in Table 2.

PLD1::mTagBFP2 displayed a bright continuous ring at the cell periphery, indicating plasma membrane localization, along with distinct punctate signals in the cytoplasmic region that partially overlapped with chlorophyll autofluorescence, suggesting possible mitochondrial association. This dual pattern, reported here for the first time in *Chlamydomonas*, implies that PLD1 may function at both the plasma membrane and intracellular organelles. PMH1::YFP, previously annotated as a plasma membrane cation transporter (ACA3) [43], showed predominant peripheral localization together with diffuse cytoplasmic signal, consistent with its known role and likely representing biosynthetic intermediates.

Among the flagellar candidates, FAP215::YFP was observed along the entire length of flagella, with a distribution broader than that of FAP178::YFP. In contrast, FAP178::YFP, a central microtubule protein identified by Gui Miao et al. [44], exhibited a narrow continuous line along flagella, matching its expected localization on the central microtubule pair. The broader signal of FAP215 suggests that it may reside in the flagellar matrix or associate with the flagellar membrane rather than being tightly restricted to the axoneme. BBS4::YFP—As a known flagellar/ciliary protein, BBS4 served as a positive control [28]. The YFP signal was detected both along the flagella (appearing as punctate or continuous distribution) and as a prominent focus near the basal body region, which is consistent with the known enrichment of BBS4 at the basal body and its dynamic trafficking within the flagellum. This localization pattern supports the reliability of our proteomic dataset and the specificity of our fluorescence assays. All localization patterns were reproducible across at least two independent transformants, and the expression of full-length fusion proteins was confirmed by Western blotting (Figure S2).

## 3. Discussion

In this study, we established a reliable pipeline for the parallel isolation of highly enriched plasma membrane and flagellar membrane from *Chlamydomonas reinhardtii* as a prerequisite for comparative proteomics. The enrichment of the fractions was supported by transmission electron microscopy and Western blotting using compartment specific markers (Figure 1, Figure 2 and Figure 3). The aqueous two-phase partitioning method originally developed by Dolle [31] and optimized by Norling et al. [30] yielded plasma membranes largely devoid of detectable of chloroplast, thylakoid and endomembrane contaminants. Our flagellar membrane isolation followed the classical deflagellation and purification strategy of Witman et al. [36], which has served as the standard for flagellar biology for decades. The stringent validation using a panel of markers provides an unprecedented level of quality control for both membrane fractions. Notably, while trace amounts of chloroplast proteins (e.g., RbcL) were detected by mass spectrometry in the flagellar membrane fraction, they remained undetectable by Western blotting (Figure 1D), reflecting the higher sensitivity of MS rather than a failure of membrane enrichment. This is a well-recognized phenomenon in high-sensitivity organellar proteomics [46,47]. Similarly, the plasma membrane-enriched fraction contained a number of mitochondrial proteins (e.g., components of the oxidative phosphorylation system). As documented in the organellar proteomics literature, complete removal of all non-target organelles is technically impossible, and “subcellular fractionation is notorious for cross-contamination” [48]. Even with highly enriched plasma membrane preparations, a small percentage of mitochondrial contaminants is typically present [49]. The challenge of distinguishing genuine organelle components from contaminants due to “the inability to generate pure fractions of an organelle” is a recognized major issue in the field [50]. Therefore, the low-level presence of mitochondrial proteins in the PM fraction does not invalidate our overall conclusion; rather, it reflects the expected background of high-sensitivity MS-based proteomics and the inherent difficulty of achieving absolutely pure subcellular fractions. The high enrichment of specific markers (FOX1, H^+^-ATPase for PM; CAV2, FMG-1 for FM) and the absence of major contaminants by Western blot (Figure 1D, Figure 2B and Figure 3C) confirm that both membrane fractions are sufficiently highly enriched for comparative proteomics.

Comparative proteomics identified 2779 proteins, with hundreds differentially enriched between the two membrane systems. The enrichment of proton transport and ATP synthesis pathways in the PM fraction underscores the plasma membrane’s central role in cellular energy coupling and ion homeostasis, which are critical for maintaining the electrochemical gradient required for nutrient uptake and signaling (Figure S1A). In contrast, the specialization of the FM fraction for cilium movement, axoneme assembly and IFT highlights the flagellar membrane as a dynamic hub for motor-driven processes and signal transduction (Figure S1B). This functional divergence is consistent with the flagellar membrane’s role as a specialized signaling platform that interfaces directly with the external environment, while the plasma membrane serves as the primary metabolic and homeostatic barrier of the cell.

The specialized composition of the flagellar membrane has long been hypothesized as a consequence of the ciliary gate mechanism [5,25,26,27,28,51]. The transition zone at the base of the cilium functions as both a selective diffusion barrier and a docking station for IFT, ensuring that ciliary membrane proteins are enriched while non-ciliary residents are excluded [52,53]. Our proteomic data provide the first quantitative, system-level view of this functional segregation. The striking enrichment of IFT components, dynein arms and flagellar-associated proteins in the flagellar membrane fraction is consistent with the absolute requirement of these proteins for flagellar motility and maintenance [12]. Conversely, the predominance of metabolic and transport-related functions in the plasma membrane reflects its role as the primary interface between the cell and its environment.

Importantly, our proteomic dataset successfully recapitulated well-established flagellar membrane proteins, including CAV2 and FMG-1, as well as IFT components. Pazour et al. performed the first high-confidence proteomic analysis of *Chlamydomonas* flagella, identifying hundreds of proteins, many of which are membrane-associated components of motor and signaling pathways [12]. The subsequent flagellar proteome database has served as a cornerstone resource for the field (https://cilia.pro/ChlamyFPv2/index.php, accessed on 6 July 2026). The fact that our independent dataset, generated from highly enriched flagellar membranes, recapitulates these established components—including CAV2, FMG-1 and multiple IFT subunits—strongly validates the quality of our sample preparation and quantitative pipeline.

Our dataset of 2779 identified proteins substantially extends previous *Chlamydomonas* membrane proteomic studies. The flagellar proteome reported by Pazour et al. (2005) identified 652 proteins from purified flagella (360 with high confidence and 292 with moderate confidence), covering 97 out of 101 previously known flagellar proteins [12]. Importantly, the vast majority of the high-confidence flagellar proteins identified by Pazour et al. are also present in our FM-enriched dataset, including IFT components (IFT46, IFT74, IFT172), radial spoke proteins (RSP1, RSP3), and dynein arm subunits (ODA1, ODA6, IDA2), confirming the broad coverage and reliability of our proteomic data.

Moreover, our FM proteome further confirmed the presence of several recently characterized flagellar membrane proteins, including a phospholipid flippase (ALA1, ALA2, ALA3) that shapes the ciliary membrane [13], the major flagellar membrane glycoprotein FMG-1A/1B that mediates force transduction during gliding motility [54], and voltage-gated calcium channels (VGCCs) that regulate photobehavioral responses at the flagella [38,55]. Additionally, we detected ARL13 and multiple BBSome subunits [56], as well as phospholipase D (PLD) [57], FAP12 and FAP138 [56]. These observations support the reliability and biological relevance of our flagellar membrane proteomic dataset, and also provide a comprehensive resource for future investigations of flagellar membrane protein functions (Table S1).

Consistent with the dynamic membrane–IFT interface, we also identified multiple IFT proteins in the FM fraction. Recent studies have shown that IFT particles physically interact with the flagellar membrane via ceramide-based lipid anchors [58] and that Ca^2+^ signals can disrupt this interaction [59], directly supporting the physiological presence of IFT components in flagellar membrane preparations.

Fluorescence localization independently validated the proteomic findings (Figure 5B). PLD1 exhibited a dual localization at the plasma membrane and punctate cytoplasmic structures, suggesting its potential involvement in membrane lipid remodeling at multiple subcellular sites. FAP215 displayed a broader flagellar signal compared to the axoneme-restricted FAP178, and subflagellar fractionation confirmed its enrichment in the flagellar membrane/matrix fraction (Figure S3), supporting its localization in the flagellar matrix or membrane, where it may participate in flagellar surface-related processes. These localization patterns provide the first subcellular distribution maps for several previously uncharacterized proteins and corroborate the proteomic enrichment data.

To our knowledge, this work represents the first systematic comparison of the plasma membrane and flagellar membrane proteomes in any eukaryotic organism. By analyzing both membrane systems from the same genetic background under identical experimental conditions, we overcame the limitations of cross-study comparisons and generated a unique resource for the field. Although it has long been recognized that the ciliary membrane is a specialized subdomain of the plasma membrane with distinct protein and lipid compositions [13,14,25], direct quantitative comparisons between these two contiguous systems have remained elusive. Previous studies characterized the flagellar proteome in isolation [8] or examined candidate proteins individually [28], while plasma membrane proteomics used different strains and conditions [30]. Our work bridges this gap by analyzing both membrane systems side-by-side under identical conditions, thereby eliminating inter-study variability and enabling meaningful quantitative comparisons.

Several limitations should be acknowledged. First, achieving complete purity of subcellular fractions is inherently challenging; however, the high enrichment of specific markers (FOX1, H^+^-ATPase for PM; CAV2, FMG-1 for FM) and the lack of detectable contaminants (PsbA, RbcL, Arf1, RSP3) by Western blot (Figure 1D, Figure 2B and Figure 3C) indicate that the fractions are suitable for comparative proteomics.

Second, membrane proteins, especially those with multiple transmembrane domains, are often poorly detected in bottom-up proteomics. Their transmembrane helices contain few lysine/arginine residues, limiting trypsin digestion and the generation of suitable peptides. In addition, hydrophobic peptides are prone to loss during sample processing, TMT labeling, and LC-MS detection [60,61,62]. Consequently, large multi-transmembrane proteins such as CAV2 (predicted 24 transmembrane segments [38]) may be identified with only a single peptide, and heavily glycosylated proteins like FMG-1 suffer from hindered digestion, suppressed ionization, and glycoform heterogeneity, leading to distorted MS quantification [63,64,65]. For both cases, Western blotting independently confirms their expected enrichment (CAV2 and FMG-1 in FM; Figure 3C). These technical limitations do not compromise the main conclusions, as the quantitative enrichment patterns of bona fide PM and FM proteins are clear and reproducible (Figure 4A–C).

Third, only two biological replicates were used per group, which limits statistical power. To mitigate this, we applied a stringent fold-change cutoff (|log_2_FC| > 1) combined with consistent regulation between replicates as the primary criterion for defining differentially enriched proteins [66,67]. Importantly, our main conclusions are independently validated by orthogonal methods (Western blotting and fluorescence localization; Figure 1, Figure 2, Figure 3 and Figure 5B).

Fourth, our quantitation provides relative rather than absolute protein abundance; absolute stoichiometry would require targeted approaches such as PRM [68]. Fifth, the analysis was performed on cells grown under standard laboratory conditions; the membrane proteome may undergo dynamic remodeling under stress or during the cell cycle [69]. Sixth, although our dataset provides a comprehensive resource, the current study did not identify novel ciliary gate proteins. While fluorescence localization validated the differential enrichment of several candidates, we have not yet pinpointed specific gatekeepers. This represents a major direction for future investigation using functional perturbations and protein mislocalization assays.

The membrane isolation protocol described here also provides a reliable basis for future comparative lipidomic studies of the plasma membrane and flagellar membrane.

In summary, this study delivers the first direct comparison of the plasma membrane and flagellar membrane proteomes in *C. reinhardtii*. The dataset reveals the functional specialization of these two contiguous membrane systems and serves as a valuable resource for future investigations into ciliary gate mechanisms, flagellar signal transduction, and the molecular basis of ciliopathies.

## 4. Materials and Methods

### 4.1. Algal Strains and Cell Culture

*C. reinhardtii* wild-type CC-125 (cw^+^) and cell-wall-deficient *cw15* (CC-400) strains, both derived from the same 137c genetic background [32,35], are routinely used in our laboratory; the temperature-sensitive mutant strain *fla10* (CC-1919; *fla10-1* allele, encoding one subunit of the anterograde IFT motor kinesin-2) was obtained from the *Chlamydomonas* Resource Center (CRC, St. Paul, MN, USA; http://www.chlamycollection.org); our laboratory initially acquired this strain from CRC in 2019 and has since maintained it [70]. All transgenic strains were grown in liquid Tris-acetate-phosphate (TAP) [6] or on solid TAP agar (1.5%) plate under continuous illumination (30 μE·m^−2^·s^−1^) at 22 °C.

For transformation, 5 × 10^7^ cells were electroporated in the presence of 200 ng of linearized plasmid using an ECM 630 electroporation system (BTX, Holliston, MA, USA), following previously described procedures [16]. After a 7-day growth period on selective TAP agar (1.5%) plate with 25 μg·mL^−1^ hygromycin (Roche, Basel, Switzerland) or 10 μg·mL^−1^ paromomycin (Sigma-Aldrich, St. Louis, MO, USA), transformants were picked and transferred to a fresh TAP plate or 96-well-plates containing 200 µL liquid TAP medium per well for further analysis.

### 4.2. Plasma Membrane Purification

Plasma membranes were isolated essentially as described previously [29]. Briefly, *C. reinhardtii cw15* cells were harvested, washed, and resuspended in a grinding buffer containing 0.5 M sucrose, 50 mM HEPES-KOH (pH 7.5), and 5 mM Ethylenediaminetetraacetic acid (EDTA). The cells were disrupted by sonication on ice, and the homogenate was centrifuged at 10,000× *g* for 15 min to remove unbroken cells and cell debris. The supernatant was then centrifuged at 48,000× *g* for 2 h to obtain a microsomal pellet [30], which was subsequently resuspended in a washing buffer (5 mM potassium phosphate buffer, pH 7.8, 0.33 M sucrose, 4 mM KCl). Plasma membranes were purified from this microsomal fraction by aqueous two-phase partitioning in a system composed of 6.4% (*w*/*w*) dextran T500 and 6.4% (*w*/*w*) polyethylene glycol 4000, containing 60 mM NaCl, 0.33 M sucrose, and 5 mM potassium phosphate (pH 7.8) [31]. After thorough mixing, the two phases were separated by centrifugation. The upper, PEG-rich phase, enriched in right-side-out plasma membrane vesicles, was collected, diluted with a suitable buffer, and collected by ultracentrifugation. The final pellet was resuspended in a storage buffer and stored at −80 °C until use.

### 4.3. Flagellar Membrane Purification

Flagellar membranes were isolated essentially as described previously [36] with modifications. Briefly, *C. reinhardtii* wild-type CC-125 cells were harvested from mid-logarithmic phase cultures by centrifugation (2500× *g*, 5 min, 22 °C). The cell pellet was gently resuspended in ice-cold HEPES buffer (10 mM HEPES, pH 7.4, 5 mM MgSO_4_, 5 mM KCl, 1 mM CaCl_2_, 5% (*w*/*v*) sucrose). Flagella were detached by pH shock-: the pH of the suspension was rapidly lowered to 4.5 by dropwise addition of 0.5 M acetic acid under vigorous stirring, maintained at pH 4.5 for 30 s, and immediately neutralized to pH 7.0 with 0.5 M KOH.

The deflagellated cell suspension was mixed with 10 mM HEPES (pH 7.2) containing 25% (*w*/*v*) sucrose at a 2:1 ratio (cells: cushion buffer, *v*/*v*). The mixture was transferred into a 50 mL centrifuge tube. Then, using a long needle, 10 mL of the same cushion buffer (10 mM HEPES, pH 7.2, 25% sucrose) was carefully injected to the bottom of the tube beneath the sample mixture to form a discrete cushion layer. The tube was centrifuged at 2500× *g* for 10 min at 4 °C. The supernatant containing crude flagella was carefully collected, and flagella were further purified by centrifugation (10,000× *g*, 10 min, 4 °C) and resuspended in HMDEK buffer (10 mM HEPES, pH 7.4, 5 mM MgSO_4_, 1 mM Dithiothreitol (DTT), 0.5 mM EDTA, 50 mM KCl).

The purified flagella were subjected to three cycles of freezing in liquid nitrogen and thawing at 37 °C to weaken the flagellar membrane. The lysate was then centrifuged at 16,000× *g* for 10 min at 4 °C, and the supernatant was collected. This step was repeated once. The combined supernatants were then centrifuged at 22,800× *g* for 30 min at 4 °C. The resulting pellet (flagellar membrane fraction) was resuspended in HMDEK buffer and stored at −80 °C. The final supernatant (matrix fraction) was also collected if needed.

### 4.4. Transmission Electron Microscopy

To examine the ultrastructure of the isolated plasma membrane and axonemes, electron microscopy was performed as described previously [71] with modifications. Purified plasma membrane vesicles were adsorbed onto glow-discharged carbon-coated copper grids, negatively stained with 2% (*w*/*v*) uranyl acetate (or 1% phosphotungstic acid, pH 7.0) for 1 min, and air-dried. Samples were examined using a Hitachi H-7650 transmission electron microscope (Hitachi High Technologies Corporation, Tokyo, Japan) operating at 80 kV.

After removal of the flagellar membrane and matrix, axonemes were fixed with 2% glutaraldehyde, post-fixed with 1% OsO_4_ in the presence of potassium ferricyanide, dehydrated through an ethanol series, transitioned to propylene oxide, and embedded in Poly/Bed 812 epoxide resin (Polysciences, Warrington, PA, USA). Ultra-thin sections (nominal thickness 55 nm) were picked up on unsupported 300-mesh copper grids, post-stained with 2% aqueous uranyl acetate and Reynolds lead citrate, and examined in the same microscope.

### 4.5. Gene Cloning and Transformation

The plasmid pHK1034, which encodes PLD1::mTagBFP2, contains the *HSP70-RBCS2* fusion promoter, the selectable marker gene *APHVII*, the *Multi-Stop* sequence, and the 3′ UTR of *RPL23*. To generate constructs expressing PMH1::YFP, FAP215::YFP, FAP178::YFP and BBS4::YFP, the corresponding coding sequences were amplified and respectively replaced IFT46::YFP in pHK214 [72], yielding the plasmids pHK1355 (PMH1), pHK1356 (FAP215), pHK1357 (FAP178) and pHK1358 (BBS4). pHK1355–pHK1357 were linearized with *Kpn*I, while pHK1354 and pHK1358 were linearized with *Not*I. All linearized plasmids were then introduced into the *fla10* mutant by electroporation.

All these genes were amplified from the CC125 genomic DNA, the primers for amplifying the *PLD1*, *PMH1*, *FAP215*, *FAP178* and *BBS4* are shown in Table 2.

### 4.6. SDS-PAGE and Immunoblotting

The procedures for SDS-PAGE and immunoblotting were performed as described previously [73]. Briefly, Protein samples were separated by SDS-PAGE using 8% or 10% polyacrylamide gels (depending on the molecular weight of the target protein), and then electrophoretically transferred onto PVDF membranes (0.45 µm pore size). The PVDF membranes were activated by brief immersion in methanol before use, and then equilibrated in transfer buffer. The transfer was performed using a wet transfer system with transfer buffer containing 25 mM Tris, 192 mM glycine, and 20% methanol, at a constant current of 300 mA for 120–180 min on ice. After transfer, the membranes were blocked with 5% non-fat milk in TBST for 1 h at room temperature, followed by overnight incubation with primary antibodies at 4 °C. After washing, the membranes were incubated with HRP-conjugated secondary antibodies and visualized using enhanced chemiluminescence (ECL). Antibodies used in this study are listed in Table 3.

### 4.7. Live-Cell Imaging

All live-cell imaging was acquired using a Leica TCS SP8 STED confocal microscope equipped with a 100× oil-immersion objective (Leica Microsystems, Wetzlar, Germany) under static conditions. Fluorescent signals of mTagBFP2 (excitation/emission 405 nm/440–500 nm) and YFP (excitation/emission 514 nm/515–560 nm) were processed using the Leica software LAS X (version 3.4.2).

### 4.8. Proteomics Sample Preparation

Membrane fraction pellets were washed three times with ice-cold acetone and resuspended in 8 M urea. Protein concentration was determined using a BCA Protein Assay Kit (Beyotime Biotechnology, Nantong, China). An aliquot of 50 µg protein was reduced with 5 mM DTT at 56 °C for 30 min, followed by alkylation with 11 mM iodoacetamide at room temperature in the dark for 15 min. The urea concentration was diluted to below 2 M. Proteins were digested with trypsin at an enzyme-to-substrate ratio of 1:50 (*w*/*w*) at 37 °C overnight, followed by a second digestion at a ratio of 1:100 (*w*/*w*) for 4 h. The resulting peptides were desalted using Strata X C18 columns (Phenomenex, Torrance, CA, USA) and vacuum-dried. Dried peptides were reconstituted in 0.5 M TEAB and labeled with TMT reagents according to the manufacturer’s instructions. In brief, TMT reagents (Thermo Fisher Scientific, Waltham, MA, USA) were dissolved in acetonitrile, mixed with peptides, and incubated at room temperature for 2 h. Labeled peptides were pooled, desalted, and vacuum-dried. The pooled sample was fractionated by high-pH reversed-phase HPLC on an Agilent 300Extend C18 column (Agilent Technologies, Santa Clara, CA, USA). Peptides were separated with a gradient of 8–32% acetonitrile (pH 9) over 60 min into 60 fractions, which were concatenated into 10 fractions. Each fraction was vacuum-dried prior to LC-MS/MS analysis.

### 4.9. LC-MS/MS Analysis

Dried TMT-labeled peptides were reconstituted in 0.1% formic acid and analyzed on a Q Exactive™ Orbitrap mass spectrometer coupled to an EASY-nLC™ 1000 system (Thermo Fisher Scientific, Waltham, MA, USA). Mobile phase A consisted of 0.1% formic acid and 2% acetonitrile in water; mobile phase B consisted of 0.1% formic acid and 90% acetonitrile in water. Peptides were separated over a 40 min gradient: 7–23% B (0–26 min), 23–35% B (26–34 min), 35–80% B (34–37 min), and 80% B (37–40 min) at a flow rate of 400 nL/min. Eluted peptides were ionized via a nanoelectrospray ionization (NSI) source at 2.0 kV. Full MS scans were acquired over a range of 350–1800 m/z at a resolution of 70,000. The top 20 most intense precursor ions were selected for HCD fragmentation at 31% normalized collision energy. MS/MS scans were acquired starting from 100 m/z at a resolution of 17,500. The AGC target was set to 5 × 10^4^, the signal threshold to 10,000 ions/s, the maximum injection time to 200 ms, and the dynamic exclusion time to 30 s.

### 4.10. Database Search

MS/MS data were searched using MaxQuant (version 2.0.3.0) against the UniProt Chlamydomonas reinhardtii protein database (14,338 sequences) supplemented with a decoy database and a common contaminant database. Trypsin/P was specified as the protease with up to 2 missed cleavages. The minimum peptide length was set to 7 amino acids, and the maximum number of modifications per peptide was 5. The precursor mass tolerances for the first search and main search were 20 ppm and 5 ppm, respectively. The fragment ion mass tolerance was 0.02 Da. Carbamidomethylation of cysteine was set as a fixed modification. Oxidation of methionine and N-terminal protein acetylation were set as variable modifications. Quantification was performed using the TMT 6-plex method. The FDR was set to 1% at both the protein and PSM levels.

### 4.11. Data Analysis

Proteins were identified and quantified as described above. Due to the limited number of biological replicates (*n* = 2 per group), differential abundance was assessed using a stringent fold-change cutoff (|log_2_FC| > 1, i.e., >2-fold difference) combined with consistent direction of regulation between the two replicates, rather than relying on *p*-values or FDR. Proteins meeting these criteria were considered differentially enriched. Gene Ontology (GO) functional enrichment and Kyoto Encyclopedia of Genes and Genomes (KEGG) pathway enrichment analyses were performed using the R package clusterProfiler (version 4.7.0), with *p* < 0.05 as the significance threshold for enrichment (based on the hypergeometric test, not on differential expression).

## 5. Conclusions

This study provides the first systematic comparison of the plasma membrane and flagellar membrane proteomes in *Chlamydomonas reinhardtii*. The two membrane systems exhibit distinct functional signatures: the plasma membrane is enriched in proton transport and ATP synthesis proteins, while the flagellar membrane specializes in cilium movement, axoneme assembly and intraflagellar transport. Fluorescence localization validated the proteomic data and revealed novel subcellular patterns. This comprehensive proteomic dataset serves as a valuable resource for future studies on ciliary gate mechanisms and protein sorting between contiguous yet functionally distinct membrane domains.

## Figures and Tables

**Table 1:** MS/MS spectrum database search analysis summary.

Total Spectrums	Matched Spetrums	Peptides	Unique Peptides	Identified Proteins	Quantifiable Proteins
119,578	26,295	15,852	15,535	2779	2287

**Table 2:** List of primers utilized in this study.

Primers	Sequence (5′ to 3′)
PLD1-F	AACATCTTAAACATATGGGTTGCGCCAGCTCCAA
PLD1-R	CTTGCTCACCATGATATCCTTGAACATGTCCCAGAGCTTGTCG
PMH1-F	CACAACAAGCCCATATGGCGGAACAGGAGAAACCCAAAG
PMH1-R	GCCGGAGGCGCCGATATCCATGTGCCGCTTCGAGGAAATC
FAP215-F	CACAACAAGCCCATATGGGCGCTGGGTGCTCGCGAAC
FAP215-R	GCCGGAGGCGCCGATATCCACGGCCTCCAGCACATTGACGAT
FAP178-F	CACAACAAGCCCATATGACCAACCGGGCGACGTTGTC
FAP178-R	GCCGGAGGCGCCGATATCCGAGGTCCCGCCTGCCAGC
BBS4-F	CACAACAAGCCCATATGTCGTCATTAGCGCAGCAGCG
BBS4-R	GCCGGAGGCGCCGATATCCATGCCCAGCAGCTGCCC

**Table 3:** List of antibodies utilized in this study.

Antibodies	Animal	Dilution Ratio	Cellular Compartment/Enrichment	Company/Resource
FOX1	Rabbit	1:1000	PM	Gift from Professor Joel Rosenbaum
H^+^-ATPase	Rabbit	1:2500	PM	Gift from Professor Joel Rosenbaum
PsbA	Rabbit	1:20,000	Chloroplast (thylakoid)	Agrisera, AS05 084
RbcL	Rabbit	1:10,000	Chloroplast (stroma)	Agrisera, AS03 037
Arf1	Rabbit	1:1000	Golgi/endosomal	Agrisera, AS08 325
RSP3	Rabbit	1:2000	Axoneme	Gift from Professor Joel Rosenbaum
CAV2	Rabbit	1:2000	FM	Gift from Professor Joel Rosenbaum
FMG-1	Mouse	1:3000	FM	Huang et al., 2007 [39]
PKD2	Rabbit	1:2000	FM	Huang et al., 2007 [39]
IFT46	Rabbit	1:2000	FM (IFT)	custom-generated in our laboratory
IFT74	Rabbit	1:2500	FM (IFT)	Gift from Professor Joel Rosenbaum
IFT27	Rabbit	1:2000	FM (IFT)	Gift from Professor Joel Rosenbaum
IP3R	Rabbit	1:2000	ER	Gift from Professor Joel Rosenbaum
Anti-Mouse IgG	Goat	1:5000	—	Sigma, A4416
Anti-Rabbit IgG	Goat	1:5000	—	Sigma, A0545

Notes: PM, plasma membrane; FM, flagellar membrane; IFT, intraflagellar transport; ER, endoplasmic reticulum; “—” indicates not applicable (secondary antibodies).

## Data Availability

The data supporting this study are available within the article and its Supplementary Materials. The mass spectrometry proteomics data have been deposited to the ProteomeXchange Consortium (https://proteomecentral.proteomexchange.org, accessed on 3 July 2026) via the iProX partner repository [74,75] with the dataset identifier PXD077643. Further inquiries can be directed to the corresponding author.

## Supplementary Materials

The following supporting information can be downloaded at: https://www.mdpi.com/article/10.3390/ijms27146141/s1.
