# Supplementary material for: Comparative Proteomics of Plasma and Flagellar Membranes in Chlamydomonas reinhardtii
Source: Int J Mol Sci. 2026 Jul 9;27(14):6141. doi: 10.3390/ijms27146141 (PMC13410224; doi:10.3390/ijms27146141)
Supplement: Supplementary file 1 [file ijms-27-06141-s001.zip › ijms-4401433-Figures S1-S3.pdf]

Supporting Information for

Comparative Proteomics of Plasma and Flagellar Membranes in *Chlamydomonas reinhardtii*

Yiwen Lin[1,2], Sheng Yao[3], Huan Long[1,*] and Kaiyao Huang[1,*]

* Correspondence: huanlong@ihb.ac.cn; huangky@ihb.ac.cn

This Word file includes:
Figures S1 to S3

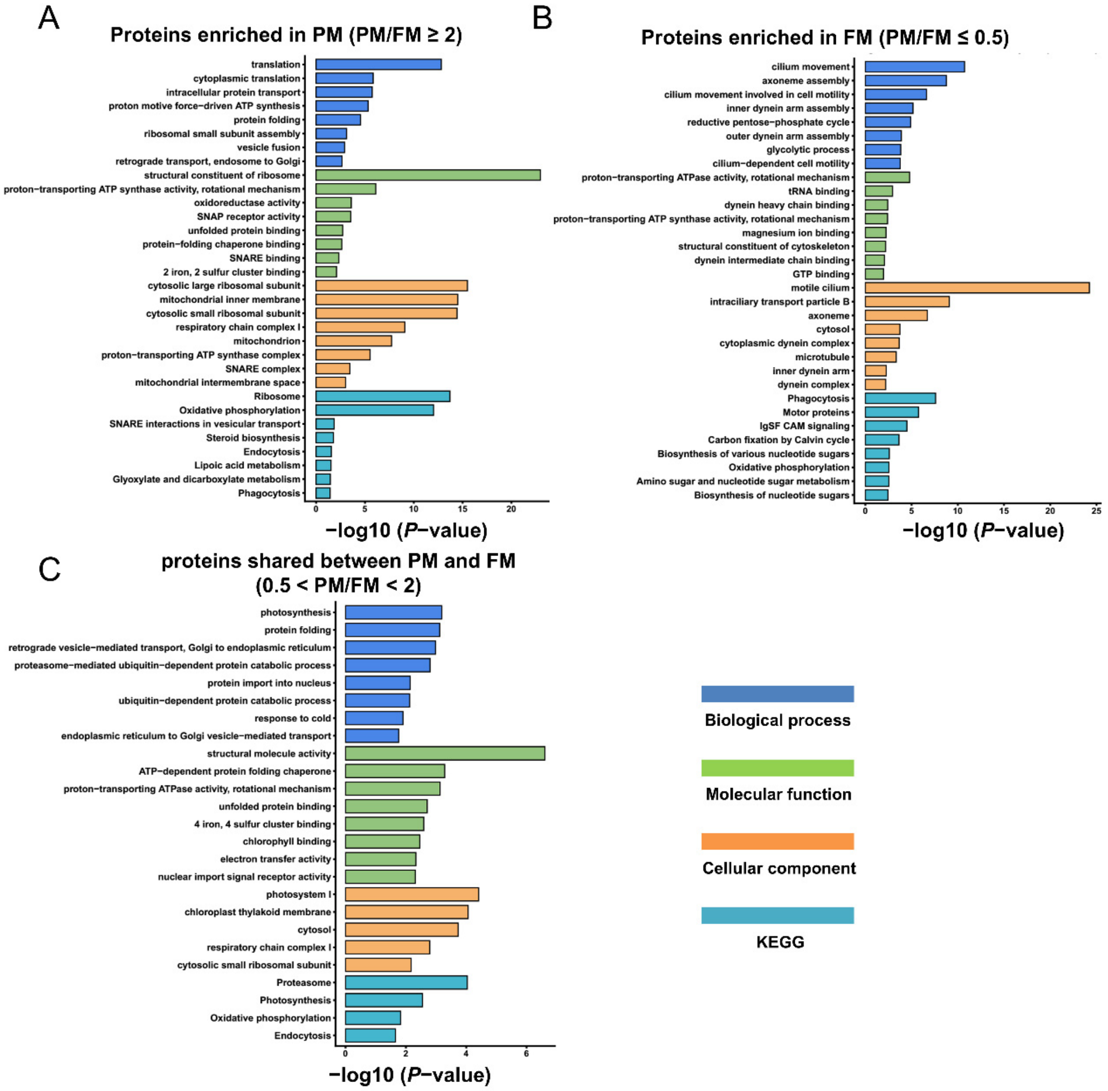

Figure S1. Comparative proteomic analysis of plasma membrane and flagellar membrane. (**A–C**) GO and KEGG enrichment analysis of (**A**) plasma membrane (PM) enriched (PM/FM ≥ 2), (**B**) flagellar membrane (FM) enriched (PM/FM ≤ 0.5) and (**C**) non-differential proteins (0.5 < PM/FM < 2), respectively. Enriched terms and pathways are indicated (fold enrichment values in parentheses).

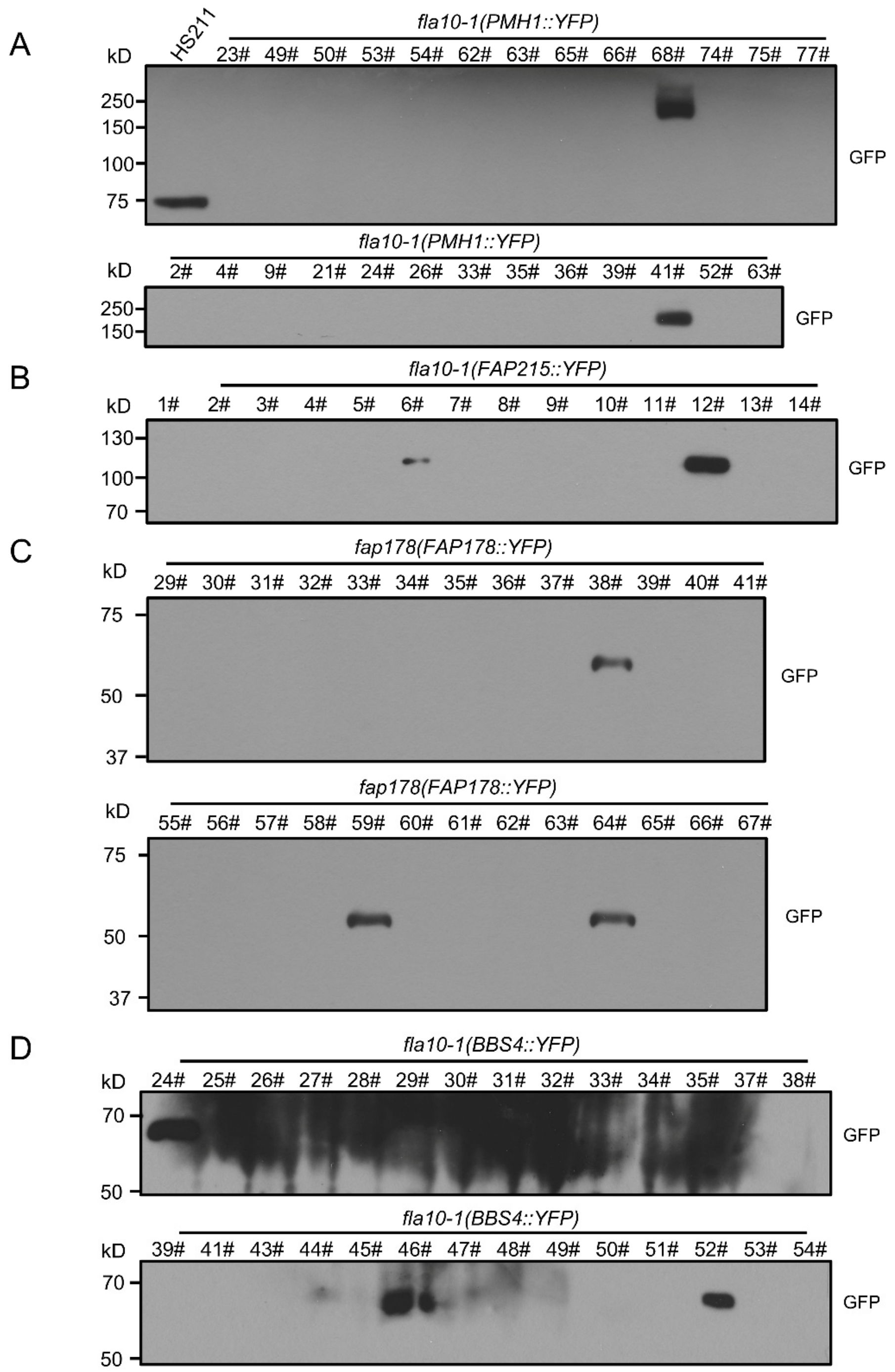

Figure S2. Western blot validation of positive transformants expressing fluorescent fusion proteins. Total protein extracts from the indicated transformants were immunoblotted with anti-GFP antibody. HS211 (IFT46::YFP, ~75 kDa) served as a positive control. (**A**) PMH1::YFP (~147 kDa), (**B**) FAP215::YFP (~97 kDa), (**C**) FAP178::YFP (~52 kDa) and (**D**) BBS4::YFP (~72 kDa) each showed a single predominant band at the expected molecular weight, confirming expression of the full-length fusion proteins. Molecular weight markers (kDa) are indicated on the left.

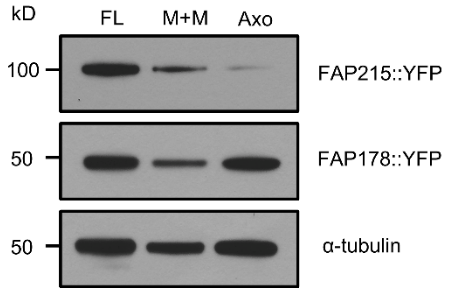

Figure S3. Subflagellar fractionation analysis of FAP215::YFP and FAP178::YFP. FAP215::YFP is enriched in the M+M fraction, whereas the central microtubule protein FAP178::YFP is enriched in the Axo fraction. Fl (flagella), M+M (membrane and matrix), and Axo (axoneme).
